# Giant Pilomatrixoma of the Scrotum: A Rare Case Presentation

**DOI:** 10.7759/cureus.22228

**Published:** 2022-02-15

**Authors:** Geet Adhikari, Gauri S Jadhav

**Affiliations:** 1 Department of Surgery, Dr. Hedgewar Hospital, Aurangabad, IND

**Keywords:** benign scrotal mass, hair follicle tumor, calcifying epithelioma of malherbe, pilomatrixoma, pilomatricoma

## Abstract

Pilomatrixoma is a benign adnexal skin tumor involving the hair follicle matrix. It is found in children, predominantly involving the face. We report here an interesting and rare case of a 52-year-old male presenting with a large, firm, and painless mass over the scrotum considered to be a sebaceous cyst. The mass was completely excised and a biopsy report revealed it to be a pilomatrixoma of the scrotum. The patient recovered well postoperatively with no recurrence over two years.

## Introduction

First described by Malherbe in 1880, pilomatrixoma is a rare, benign, slow-growing, cutaneous tumor of the hair follicle matrix. It is also called calcifying epithelioma of Malherbe [1]. It is more common in the pediatric age group and women; however, the overall incidence is roughly one in 800 cutaneous tumors only [2]. Few retrospective studies have also reported a bimodal distribution of presentation with the first peak around the first decade of life and a second peak at the sixth decade [3]. It is commonly located over the face (preauricular, periorbital, neck, and upper trunk) [4]. It is mostly a solitary and asymptomatic lesion, whereas multiple forms have syndromic associations. A proliferating variant was reported first in 1997 exhibiting high mitosis and cellular atypia mimicking a malignancy; however, chances of a true malignancy are rare [5].

The adnexal tumor is often misdiagnosed clinically due to its resemblance with other common skin masses like sebaceous, epidermoid, or dermoid cysts. The diagnostic modality of choice is tissue biopsy, which shows the presence of characteristic basaloid cells and mummified shadow cells with areas of calcification amidst shadow cells [6]. S100 proteins have been studied in detail for their role as biochemical markers for pilomatrixoma [7]. There is no role of medical therapy as the tumor does not exhibit spontaneous regression; surgical removal is the only option by all means.

Here, we present a case of scrotal mass that was initially considered to be a sebaceous cyst but turned out to be a pilomatrixoma of the scrotum. Only one such case report has been published before.

## Case presentation

**Figure 1:**
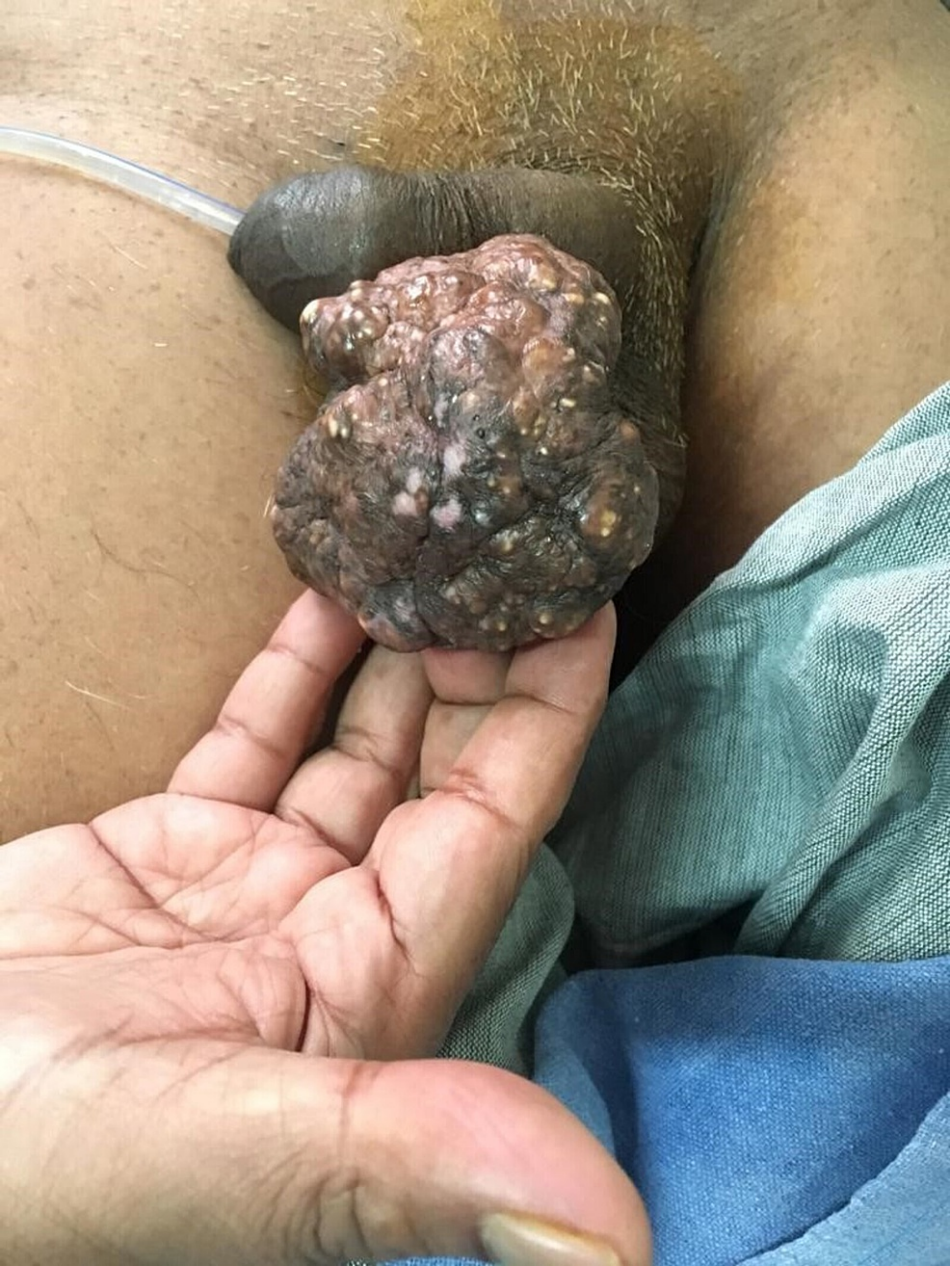
Giant scrotal mass on inspection.

A 52-year-old male presented to the surgery outpatient department with a chief complaint of large scrotal mass over the scrotum for one year. The onset was insidious and gradually progressive in size over the duration causing increasing discomfort. There was no complaint of associated fever or pain. Bowel habits were regular. The patient was also diagnosed with urethral stricture one week ago for which a urethral catheterization was done elsewhere. There was no history of systemic illness. On examination, a large, firm, non-tender, pear-shaped, sessile mass was noted on the anterior aspect of the scrotum with irregular margins and lobulated appearance (Figure 1).

**Figure 2:**
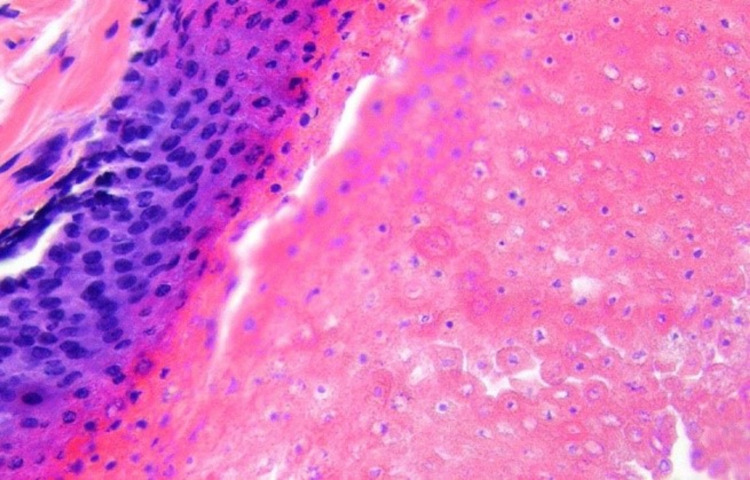
Biopsy report suggestive of a pilomatrixoma.

Bilateral testes were normal and could be felt separately. No regional lymphadenopathy was noted. A diagnosis of a scrotal sebaceous cyst was considered. The patient was posted for surgery under spinal anesthesia. Mass was excised in toto and sent for histopathological examination. The scrotum was closed in layers with absorbable sutures. The patient was discharged in clinically stable condition on the next day. The histopathological report, to our surprise, was suggestive of a pilomatrixoma of the scrotum (Figure 2).

## Discussion

Pilomatrixoma commonly is a single, nodular, painless mass, adhered to the underlying skin but not extending into the deeper planes [8]. Based on appearance, a pilomatrixoma lesion can be classified into five clinical types: protruding mass, pigmented, mixed type, ulcerated, and keloid-like. The mass type is a predominant presentation in the available literature [9]. Four stages of the histomorphological pattern were explained by Kaddu et al. as early, fully developed, early regressive, and late regressive stages [5].

The beta-catenin gene (CTNNB1), which plays a vital role in the development of hair follicles, is mutated in up to 75% of cases. The presence of proto-oncogene B-cell lymphoma antigen 2 (BCL2) has also been associated with pilomatrixoma. Multiple forms have been observed to be associated with familial conditions such as Gardner syndrome, myotonic dystrophy, sarcoidosis, Steinert’s disease, or Turner syndrome [10]. The risk of malignancy is rare; however, locally aggressive pilomatrix carcinoma with distant metastasis has been reported in studies before [11,12]. Differential diagnoses are sebaceous cyst, epidermal cyst, dermoid cyst, sebaceous adenoma or carcinoma, capillary hemangioma, squamous cell tumor, etc. [13].

Imaging modalities like ultrasound (USG), CT, and MRI scans have all been used to support the diagnosis. USG is acceptable enough in the majority of the cases with a positive predictive value of 95.5% since it is quick, cheap, and easily available. The definitive diagnosis of pilomatrixoma is, however, made by histopathological examination [9]. On histology, the tumor is well-defined, comprising of peripheral basaloid cells proliferation and centrally located structureless eosinophilic cells, which lack nuclei called shadow cells or ghost cells typical of trichilemmal keratinization. Areas of calcification can be seen within the region of shadow cells [14].

The treatment of pilomatrixoma is essentially complete surgical resection, with 5 to 10 mm of safety margins. With a well-performed surgical procedure, the rate of recurrence is low at around 1.5% [15]. Mohs micrographic surgery is now being used to ensure complete margin-free excision, especially in cases of suspected malignancy [16].

## Conclusions

Pilomatrixoma of the scrotum is a very rare entity. It is often misdiagnosed due to high clinical polymorphism. An ultrasound examination of a suspicious scrotal mass should be done to support the preoperative diagnosis. Histopathological evidence is confirmatory and the treatment is complete surgical excision with extremely low chances of recurrence.
